# Early Cognitive and Behavioral Changes in Primary Lateral Sclerosis: A Population‐Based Study

**DOI:** 10.1111/ene.70720

**Published:** 2026-07-31

**Authors:** Andrea Calvo, Cristina Moglia, Antonio Canosa, Umberto Manera, Rosario Vasta, Enrico Matteoni, Alessandra Maccabeo, Sara Cabras, Giorgio Pellegrino, Emilio Minerva, Anastasia Dei Giudici, Vita Passidomo, Francesca Palumbo, Barbara Iazzolino, Gabriele Mora, Adriano Chiò

**Affiliations:** ^1^ ‘Rita Levi Montalcini’ Department of Neuroscience, ALS Center University of Torino Turin Italy; ^2^ Neurology 1 Azienda Ospedaliero‐Universitaria Città Della Salute e Della Scienza of Torino Turin Italy; ^3^ Institute of Cognitive Sciences and Technologies, National Research Council Rome Italy

## Abstract

**Objective:**

Primary lateral sclerosis (PLS) is a rare upper motor neuron neurodegenerative disorder whose cognitive profile, particularly at early stages, remains incompletely defined. We aimed to characterize cognitive and behavioral features of PLS at diagnosis and compare them with predominant upper motor neuron amyotrophic lateral sclerosis (PUMN‐ALS) and healthy controls (HCs).

**Methods:**

Patients diagnosed with PLS between 2007 and 2021 were identified from the population‐based Piemonte and Valle d'Aosta ALS Register. Diagnoses were established according to consensus criteria, including early, probable, and definite PLS. All patients underwent comprehensive neuropsychological and behavioral assessment within 3 months of their first ALS center visit. Cognitive–behavioral status was classified using ALS–frontotemporal dementia (FTD) consensus criteria.

**Results:**

Thirty‐two PLS patients were included (mean disease duration, 25 months). Cognitive and/or behavioral impairment was identified in 29.3% of patients, most commonly affecting executive function, memory, and social cognition, including 21.1% early PLS. Compared with HCs, PLS patients showed poorer performance across several cognitive domains and higher anxiety and depression scores. Compared with matched PUMN‐ALS patients, PLS patients demonstrated slightly worse executive performance, while the overall frequency of cognitive–behavioral impairment was similar. Behavioral profiles differed qualitatively, with apathy more frequent in PUMN‐ALS. No PLS patient met criteria for frontotemporal dementia.

**Interpretation:**

Cognitive and behavioral impairments are already detectable at the time of diagnosis in a substantial proportion of patients with PLS, including early PLS, supporting the view of PLS as a multidimensional neurodegenerative disorder with early extramotor involvement.

## Graphical abstract

In this population‐based study drawn from the Piemonte and Valle d'Aosta ALS Register (2007–2021), 32 patients with primary lateral sclerosis (PLS) underwent comprehensive neuropsychological and behavioral assessment at diagnosis (mean disease duration 25 months) and were compared with matched predominant upper motor neuron ALS (PUMN‐ALS) patients and healthy controls. Cognitive and/or behavioral impairment was identified in 29.3% of PLS patients overall and in 21.1% of early PLS patients, most commonly affecting executive function, memory, and social cognition; no PLS patient met criteria for frontotemporal dementia. Compared with healthy controls, PLS patients performed worse across several cognitive domains and had higher anxiety and depression scores. Compared with matched PUMN‐ALS patients, PLS patients showed slightly worse executive performance, while apathy was more frequent in PUMN‐ALS. These findings indicate that cognitive and behavioral impairment is already detectable at the time of diagnosis in a substantial proportion of PLS patients, supporting the view of PLS as a multidimensional neurodegenerative disorder with early extramotor involvement.

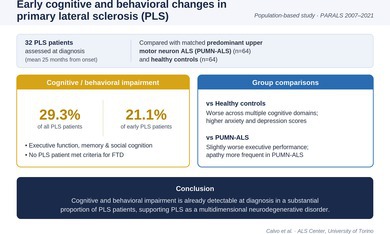

## 1 Introduction

Primary lateral sclerosis (PLS) is an adult‐onset neurodegenerative disorder characterized by selective involvement of the upper motor neurons (UMNs). Its etiology remains unknown. Compared with amyotrophic lateral sclerosis (ALS), including the predominant upper motor neuron phenotype (PUMN‐ALS), PLS is associated with a more favorable prognosis [1, 2, 3, 4]. Diagnostic criteria for PLS have been formalized only recently [5]. According to these criteria, a diagnosis of definite PLS requires symptom onset at ≥ 25 years of age, evidence of upper motor neuron dysfunction in at least two of three regions (lower limbs, upper limbs, or bulbar), and the absence of significant active lower motor neuron (LMN) degeneration for at least 4 years after symptom onset. Probable PLS is defined by the absence of LMN involvement for 2–4 years. More recently, the category of early PLS has been proposed for patients with a symptom duration of < 2 years at first evaluation who subsequently fulfill PLS diagnostic criteria during follow‐up [6].

Importantly, these criteria do not incorporate laboratory markers of UMN dysfunction, such as neuroimaging, neurophysiological, or neurochemical biomarkers, as their clinical validation remains incomplete [5, 7]. The criteria also recognize the possibility of cognitive and behavioral impairment in PLS, albeit at a lower frequency than in ALS. Consequently, the diagnosis of definite PLS is often delayed, even among patients who are referred early to tertiary ALS centers. Moreover, distinguishing PLS from PUMN‐ALS continues to pose a substantial clinical challenge [1].

Until recently, few studies have specifically examined the cognitive features of PLS, and most available evidence comes from patients assessed 5–15 years after symptom onset [8, 9]. Although a subset of individuals with PLS may develop cognitive and behavioral impairments over time [8, 10, 11], little is known about their cognitive status in the early stages of the disease. Such information is crucial for improving early diagnostic accuracy and for distinguishing PLS from PUMN‐ALS.

The aim of this study was to characterize the cognitive profile of patients with PLS at the time of diagnosis and to compare it with individuals with PUMN‐ALS and healthy subjects.

## 2 Methods

The study population comprised all patients with PLS recorded in the prospective Piemonte and Valle d'Aosta Register for ALS (PARALS) between 2007 and 2021. PARALS is a population‐based, prospective registry that has been active since 1995 in two regions of Northern Italy; its design and methodology have been described in detail previously [4, 12]. Descriptive epidemiological data on PLS in these regions have recently been published [4]. The diagnosis of PLS was established according to the Primary Lateral Sclerosis Consensus Diagnostic Criteria [5]. Patients with a disease duration between 2 and 4 years were classified as probable PLS, those with a disease duration exceeding 4 years were classified as definite PLS; finally, patients who, on their first visit, had a symptom duration of < 2 years but subsequently fulfilled the diagnostic criteria for PLS during follow‐up, were classified as early PLS [6]. For the present study, only PLS patients who underwent a comprehensive cognitive assessment (see below) were included.

We compared the cognitive and behavioral characteristics of PLS patients with those diagnosed with PUMN‐ALS. The diagnosis of PUMN‐ALS was based on internationally recognized operative diagnostic criteria [1]. At the time of diagnosis, we gathered patients' demographic and clinical information, including their scores on the ALS Functional Rating Scale–Revised (ALSFRS‐R).

Individuals with conditions that could affect cognition (e.g., intellectual disability, major stroke, or severe head injury), as well as those with alcohol or substance dependence, major psychiatric disorders, or treatment with high‐dose psychoactive medications, were excluded from the analysis. Patients who were not native Italian speakers were assessed only through an unstructured interview and were therefore also excluded.

Age‐ and sex‐matched control participants were assessed using the same test battery. Controls were recruited from residents of retirement homes or from non‐consanguineous relatives of the patients.

### 2.1 Neuropsychological Assessment

Patients and controls underwent a comprehensive neuropsychological battery assessing executive function, verbal and visual memory, attention and working memory, visuospatial abilities, language, social cognition, and behavior. The assessment was administered within 2 months of the initial visit to the ALS center. The tests were selected according to the Diagnostic Criteria for the Behavioral variant of Frontotemporal Dementia [13], and ALS‐FTD Consensus Criteria (ALSFTD‐CC) [14]. The list of tests and their classification according to the main neuropsychological domain is reported in the Table S1 [15, 16, 17].

Behavioral impairment was assessed with ECAS Behavior score (ECAS‐BS) and Frontal Systems Behavior Scale (FrSBe). ECAS Behavior score (ECAS‐BS) cut‐off values are based on the Italian validation papers [18, 19].

According to the ALSFTD‐CC [14] PLS patients were classified into five categories: (1) patients with normal cognition (PLS‐CN); (2) patients with isolated cognitive impairment (PLSci), that is, patients with evidence of executive and/or language dysfunction. Executive impairment was defined as impaired verbal fluency (letter) and/or impairment on two other nonoverlapping measures of executive functions; (3) patients with isolated behavioral impairment (PLSbi), characterized by apathy with or without other behavioral changes; (4) patients with both cognitive and behavioral impairment (PLScbi), meeting the criteria for both PLSci and PLSbi; and (5) patients with frontotemporal dementia (FTD).

### 2.2 Statistical Methods

Comparisons between tests were performed using scores corrected for age, sex, and education. As most cognitive test scores were not normally distributed, group differences were assessed using the Mann–Whitney U test. Given differences in age, sex, and site of onset among PLS, PUMN‐ALS, and control groups, comparisons involving PLS were adjusted for these variables using propensity score matching, as implemented in the MatchIt package (version 4.1.0), with a 2:1 matching ratio. The resulting matched groups were balanced across all considered variables. All statistical analyses were conducted using SPSS version 29.0 (SPSS Inc., Chicago, IL, USA).

### 2.3 Standard Protocol Approvals, Registrations, and Patient Consents

The study was approved by the Ethics Committee of the ALS Expert Center of Torino (Comitato Etico Territoriale Azienda Ospedaliero‐Universitaria Città della Salute e della Scienza, Torino, #0036344, #0038876, and #0064510), in accordance with the Italian law 2018/3 and the Legislative Decree of 14 May 2019, No. 52. Patients and controls provided written informed consent before enrollment. The database was anonymized according to Italian law for the protection of privacy (article 110 of Legislative Decree No. 196/2003, as amended in 2024).

## 3 Results

Between 2007 and 2021, a total of 57 patients were diagnosed with PLS in the Piemonte and Valle d'Aosta regions. Of these, 32 (56.1%) underwent a complete neuropsychological battery within 3 months of the first visit to the ALS center. Patients who underwent neuropsychological examination differed from those who did not in that they were younger (60.5 vs. 67.2 years, *p* = 0.02) and more frequently male (80.0% vs. 50.4%, *p* = 0.04) (Table S2). The final cohort included 16 male and 16 female, with a mean age at onset of 60.5 years (SD 8.5). The site of onset was spinal in 29 cases (90.2%) (Table 1). The mean time from onset to diagnosis was 25.2 (SD 14.3; median 21.6, range 7–69 months). Three patients at the time of the first presentation to the ALS center met the criteria for the diagnosis of definite PLS and 10 of probable PLS. The remaining 19 cases had a disease duration under 24 months and therefore were classified as early PLS. All these patients have been followed up after the diagnosis and none of them developed ALS during a follow‐up ranging from 12 to 20 years [4]. PLS and PUMN‐ALS did not differ for any clinical characteristic but for a higher monthly loss of ALSFRS‐R points in PUMN‐ALS (0.73 vs. 0.39, *p* = 0.002).

**TABLE 1:** Comparison of demographic and clinical characteristics of PLS, predominant upper motor neuron ALS (PUMN‐ALS), and healthy controls (HCs).

	PLS	PUMN‐ALS	HCs	*p*	*p*
	*N* = 32	*N* = 64	*N* = 64	PLS vs. PUMN‐ALS	PLS vs. HCs
Age at test (years, SD)	60.5 (8.5)	60.0 (12.1)	60.3 (8.3)	0.85	0.98
Education (years, SD)	10.2 (3.6)	10.8 (4.2)	11.0 (4.0)	0.49	0.34
Sex (female)	16 (50.4%)	32 (50.4%)	32 (50.4%)	1	1
Onset to test time (months, SD)	24.6 (19.5)	19.5 (16.2)	—	0.18	—
Site of onset (spinal)	29 (90.6%)	61 (95.3%)	—	0.93	—
ALSFRS‐R score	40.6 (4.5)	38.5 (5.5)	—	0.08	—
∆ALSFRS‐R (points/month),^a^ SD	0.38 (0.30)	0.73 (0.54)	—	0.002	—

^a^ (∆ALSFRS‐R) (ALSFRS‐R mean monthly decline) was calculated using the following formula: (48 – *ALSFRS‐R score at diagnosis*)/(*months from onset to diagnosis*).

### 3.1 Cognitive and Behavioral Characteristics of PLS Patients

At the time of the neuropsychological examination, 10 patients (29.3%) showed cognitive and/or behavioral impairment (3 PLSbi, 6 PLSci, and 1 PLScbi) (Table 2). Among the three patients with definite PLS, two were cognitively normal and one met criteria for PLSbi. Of the 10 patients with probable PLS, five were cognitively normal and five had PLSci. Among the 19 patients with early PLS, 15 were cognitively normal, while one had PLSci, two had PLSbi, and one had PLScbi (Table S3) (*p* = 0.11).

**TABLE 2:** Comparison of the cognitive classification of PLS and predominant upper motor neuron ALS (PUMN‐ALS) (*p* = 0.15).

	PLS (*n* = 32)	PUMN‐ALS (*n* = 64)
CN	22 (71.7%)	44 (68.8%)
PLSbi/ALSbi	3 (9.4%)	10 (15.6%)
PLSci/ALSci	6 (18.8%)	3 (4.7%)
PLScbi/ALScbi	1 (3.1%)	6 (9.4%)
FTD	0	1 (1.5%)

Abbreviations: CN, cognitively normal; FTD, frontotemporal dementia; PL/ALScbi, cognitive‐behavioral impairment; PLS/ALSbi, behavioral impairment; PLS/ALSci, cognitive impairment.

### 3.2 Comparison of the Cognitive Characteristics of PLS Patients With HCs (Table 3)

**TABLE 3:** Comparison of cognitive test results between PLS and healthy controls (HCs).

	PLS	HCs	*p*
	*N* = 32	*N* = 64	
MMSE	27.5 (26.0–29.1) *N* = 30	30 (28.7–30.0) *N* = 60	0.001
FAS	28.9 (21.4–34.3) *N* = 30	35.5 (27.2–40.2) *N* = 60	0.016
CAT	16.0 (14.3–24.5) *N* = 30	19.8 (17.3–23.1) *N* = 60	0.113
FAB	15.6 (13.0–17.2) *N* = 29	16.5 (15.1–18.0) *N* = 60	0.208
Digit Span FW	5.6 (5.0–6.0) *N* = 29	7 (6.3–8.0) *N* = 60	0.001
Digit Span BW	3.8 (2.9–4.3) *N* = 29	4.8 (4.0–5.9) *N* = 60	0.001
TMT A	40 (28–58) *N* = 26	38.5 (25.3–46) *N* = 60	0.360
TMT B	77 (45.5–141) *N* = 26	62.5 (36.3–96.3) *N* = 60	0.250
TMT B‐A	34 (10.5–107) *N* = 26	27 (10–55) *N* = 60	0.136
RAVL‐IR	37.3 (32.2–43.6) *N* = 24	43.6 (39.1–50.8) *N* = 60	0.021
RAVL‐DR	7.8 (5.8–9.8) *N* = 24	9.1 (8.2–10.8) *N* = 60	0.032
BSRT‐IR	6.0 (5.4–7.0) *N* = 17	6.4 (5.6–7.5) *N* = 60	0.856
BSRT‐DR	6.5 (5.3–8.0) *N* = 17	7.2 (6.3–8.0) *N* = 60	0.195
ROCF‐IR	31.4 (28.5–35.0) *N* = 23	35.5 (32.6–36.0) *N* = 60	0.001
ROCF‐DR	11.3 (7.2–13.0) *N* = 23	17.6 (13.9–21.2) *N* = 60	0.001
Clock	5 (3–5) *N* = 27	5 (4–5) *N* = 60	0.16
CPM47	28.8 (24.8–31.4) *N* = 27	32.2 (29.9–33.9) *N* = 56	0.001
SET‐IA	5.9 (5–6) *N* = 12	6 (5.2–6) *N* = 16	0.642
SET‐CI	4.9 (4.0–5.3) *N* = 12	5.2 (4.8–6) *N* = 16	0.099
SET‐EA	4.8 (4–6) *N* = 12	5.4 (5.1–6) *N* = 16	0.080
SET‐GS	15 (14.2–16.0) *N* = 12	16.2 (15.1–17.4) *N* = 16	0.048
HADS‐A	8.5 (6.0–12.5) *N* = 27	6.5 (4–7) *N* = 56	0.005
HADS‐D	4.0 (3.0–8.0) *N* = 27	3 (1–5) *N* = 56	0.014
ECAS language	27 (22–28) *N* = 22	27 (23–28) *N* = 24	0.833
ECAS verbal fluency	16 (11–18) *N* = 22	21 (16.5–24) *N* = 24	0.001
ECAS executive	34 (27–39) *N* = 22	43 (32–44) *N* = 24	0.001
ECAS memory	18 (16–20) *N* = 22	21 (19–23.5) *N* = 24	0.004
ECAS visuospatial	12 (11–12) *N* = 22	12 (11.5–12) *N* = 24	0.620
ECAS ALS‐specific	74 (67–79) *N* = 22	91 (84.3–87.8) *N* = 24	0.001
ECAS ALS nonspecific	29 (28–33) *N* = 22	33 (28–34) *N* = 24	0.011
ECAS total score	103 (96–108.5) *N* = 22	124 (110–131) *N* = 24	0.001

Abbreviations: BSRT, babcock story recall test; BW, backward; CAT, category fluency test; Clock, clock drawing test; CPM47, Raven's colored progressive matrices; DR, delayed recall; ECAS, Edinburgh cognitive and behavioral ALS screen; FAB, frontal assessment battery; FAS, letter fluency test; FW, forward; HADS‐A, Hospital Anxiety and Depression Scale‐ Anxiety; HADS‐D, Hospital Anxiety and Depression Scale‐ Depression; IR, immediate recall; MMSE, mini mental state examination; RAVL, Rey auditory verbal learning test; ROCF, Rey‐Osterrieth complex figure test; SET, story‐based empathy task; TMT, trail making test.

Compared to healthy controls, PLS patients had worse performance in most tests, including MMSE (*p* = 0.001), FAS (*p* = 0.016), Digit Span Forward (*p* = 0.001), Digit Span Backward (*p* = 0.001), RAVL‐IR (*p* = 0,021), RAVL‐DR (*p* = 0.032), ROCF‐IR (*p* = 0.001), ROCF (*p* = 0.001), CPM47 (*p* = 0.001), SET‐GS (*p* = 0.048), ECAS Verbal Fluency (*p* = 0.001), ECAS Executive (*p* = 0.001), ECAS Memory (0 = 0.004), ECAS ALS‐specific (*p* = 0.001), ECAS ALS nonspecific (*p* = 0.0.11), and ECAS total score (*p* = 0.001). In addition, they showed worse scores in HADS‐A (*p* = 0.005) and HADS‐D (*p* = 0.014). Similar results were obtained considering only early PLS patients (data not shown).

### 3.3 Comparison of the Cognitive and Behavioral Characteristics of PLS Patients With Those of PUMN‐ALS (Table 4)

**TABLE 4:** Comparison of cognitive test results between PLS and PUMN‐ALS.

	PLS	PUMN‐ALS	*p*
	*n* = 32	*n* = 64	
MMSE	27.5 (26.0–29.1) *N* = 30	28.9 (27.0–30.0) *N* = 64	0.006
FAS	28.9 (21.4–34.3) *N* = 30	30.9 (23.2–38.2) *N* = 62	0.234
CAT	16.0 (14.3–24.5) *N* = 30	20.3 (17.0–24.4) *N* = 62	0.075
FAB	15.6 (13.0–17.2) *N* = 29	15.6 (13.0–17.2) *N* = 56	0.752
Digit Span FW	5.6 (5.0–6.0) *N* = 29	5.8 (5.0–6.6) *N* = 61	0.256
Digit Span BW	3.8 (2.9–4.3) *N* = 29	4.0 (3.3–4.5) *N* = 61	0.132
TMT A	40 (28–58) *N* = 26	41 (25–52) *N* = 57	0.578
TMT B	77 (45.5–141) *N* = 26	62 (38–124) *N* = 57	0.305
TMT B‐A	34 (10.5–107) *N* = 26	28 (8–68) *N* = 57	0.543
RAVL‐IR	37.3 (32.2–43.6) *N* = 24	41.1 (35.0–51.0) *N* = 46	0.105
RAVL‐DR	7.8 (5.8–9.8) *N* = 24	9.0 (6.7–11.6) *N* = 46	0.099
BSRT‐IR	6.0 (5.4–7.0) *N* = 17	6.5 (4.8–8.0) *N* = 37	0.773
BSRT‐DR	6.5 (5.3–8.0) *N* = 17	7.5 (6.3–8.0) *N* = 37	0.106
ROCF‐IR	31.4 (28.5–35.0) *N* = 23	32.1 (29.6–35.4) *N* = 51	0.435
ROCF‐DR	11.3 (7.2–13.0) *N* = 23	13.4 (8.2–14.4) *N* = 51	0.056
Clock	5 (3–5) *N* = 27	5 (4–5) *N* = 53	0.196
CPM47	28.8 (24.8–31.4) *N* = 27	29.2 (25.5–31.8) *N* = 62	0.966
SET‐IA	5.9 (5–6) *N* = 12	5 (4.1–6) *N* = 21	0.699
SET‐CI	4.9 (4.0–5.3) *N* = 12	5.1 (4.0–6) *N* = 21	0.152
SET‐EA	4.8 (4–6) *N* = 12	4.9 (4.0–5.6) *N* = 21	0.699
SET‐GS	15 (14.2–16.0) *N* = 12	15 (12.7–16.1) *N* = 21	0.839
HADS‐A	8.5 (6.0–12.5) *N* = 27	7.5 (5–11) *N* = 56	0.339
HADS‐D	4.0 (3.0–8.0) *N* = 27	5 (2.3–7.0) *N* = 56	0.629
ECAS language	27 (22–28) *N* = 22	27 (24–27) *N* = 33	0.405
ECAS verbal fluency	16 (11–18) *N* = 22	18 (16–20) *N* = 33	0.045
ECAS executive	34 (27–39) *N* = 22	37 (32–40) *N* = 33	0.048
ECAS memory	18 (16–20) *N* = 22	19 (17–20) *N* = 33	0.567
ECAS visuospatial	12 (11–12) *N* = 22	11 (11–12) *N* = 33	0.791
ECAS ALS‐specific	74 (67–79) *N* = 22	80 (76–86) *N* = 33	0.012
ECAS ALS nonspecific	29 (28–33) *N* = 22	30 (8–32) *N* = 33	0.677
ECAS total score	103 (96–108.5) *N* = 22	111 (102–116) *N* = 33	0.018

Abbreviations: BSRT, babcock story recall test; BW, backward; CAT, category fluency test; Clock, clock drawing test; CPM47, Raven's colored progressive matrices; DR, delayed recall; ECAS, Edinburgh cognitive and behavioral ALS screen; FAB, frontal assessment battery; FAS, letter fluency test; FW, forward; HADS‐A, Hospital Anxiety and Depression Scale‐ Anxiety; HADS‐D, Hospital Anxiety and Depression Scale‐ Depression; IR, immediate recall; MMSE, mini mental state examination; RAVL, Rey auditory verbal learning test; ROCF, Rey‐Osterrieth complex figure test; SET, story‐based empathy task; TMT, trail making test.

When comparing individual test scores, PLS patients performed significantly worse on the MMSE (*p* = 0.006), the ECAS Verbal Fluency subscore (*p* = 0.045), the ECAS Executive subscore (*p* = 0.048), the ECAS ALS‐specific score (*p* = 0.012), and the ECAS total score (*p* = 0.018), indicating slightly reduced executive functioning in the PLS group. No significant differences were observed in HADS‐A or HADS‐D scores.

**FIGURE 1:**
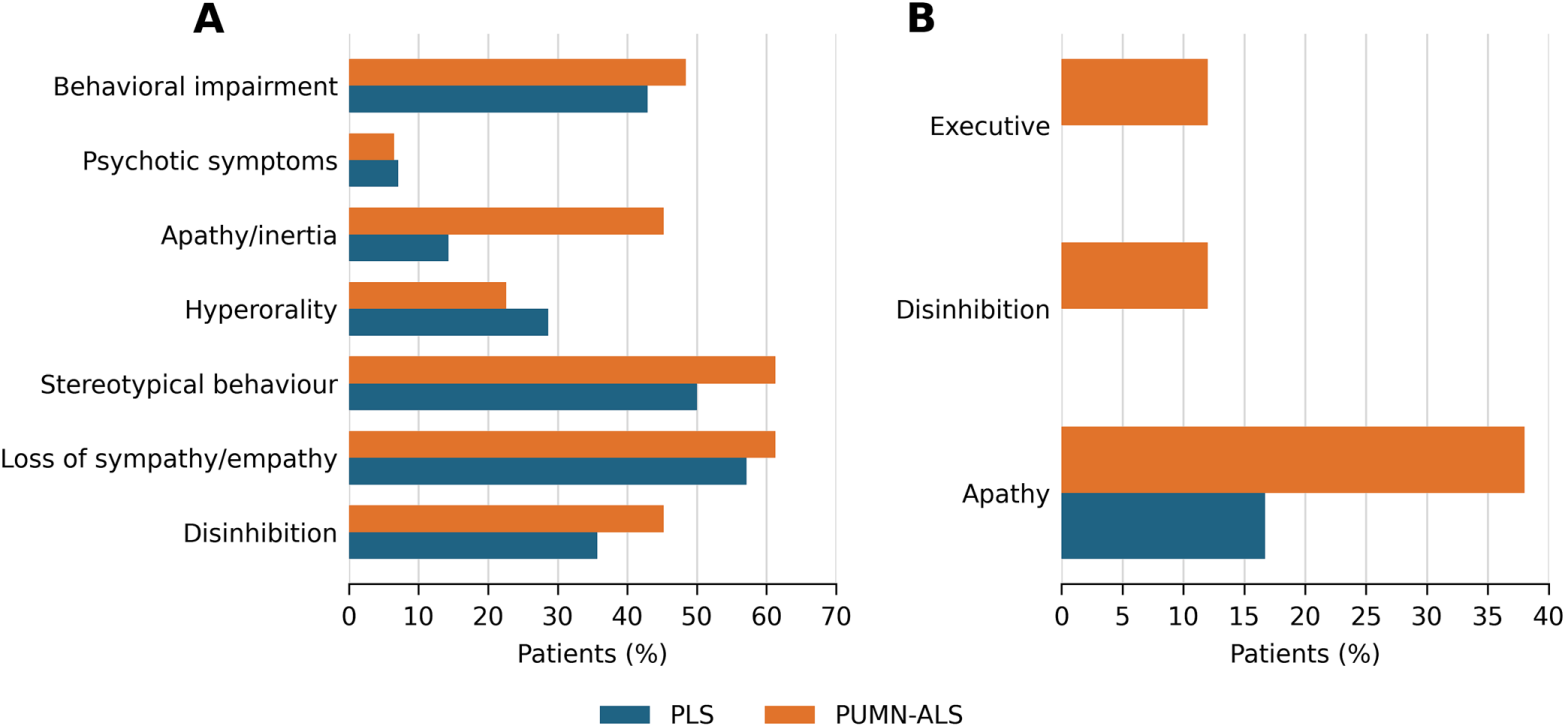
Comparison of behavioral test results between PLS and predominant upper motor neuron ALS (PUMN‐ALS). (A) ECAS Behavioral screening (PLS, 14 cases; PUMN‐ALS, 31 cases); (B) Frontal Systems Behavior Scale (FrSBe) (PLS, 18 cases; PUMN‐ALS, 50 cases).

The ECAS‐Behavioral Screen (ECAS‐BS) was completed by 14 PLS and 31 PUMN‐ALS patients. Behavioral impairment was present in 6 PLS patients (42.9%) and 15 PUMN‐ALS patients (48.4%) (*p* = 0.73). However, apathy was significantly more frequent in the PUMN‐ALS group (*p* = 0.04; Figure 1). Similar results were obtained considering only early PLS patients (data not shown).

The Frontal Systems Behavior Scale (FrSBe) was administered to 18 PLS and 50 PUMN‐ALS patients. Apathy was identified in 3 PLS patients (16.7%) and in 19 PUMN‐ALS patients (38%) (*p* = 0.19). None of the PLS patients showed disinhibition or executive behavioral disturbances, whereas 6 PUMN‐ALS patients (12%) presented with either disinhibition and/or executive behavioral impairment. Similar results were obtained considering only early PLS patients (data not shown).

The frequency of cognitive–behavioral diagnoses according to the ALSFTD‐CC [14] did not differ between PLS and PUMN‐ALS patients (*p* = 0.15) (Table S4). Notably, one patient with PUMN‐ALS was classified as having FTD, whereas no PLS patients met criteria for FTD.

## 4 Discussion

In this population‐based study, we assessed the cognitive profile of patients with primary lateral sclerosis (PLS) at the time of their initial evaluation at an ALS Expert Center. Cognitive and/or behavioral impairment, classified according to ALSFTD‐CC criteria, was identified in 29.3% of patients, with predominant involvement of executive function, spatial and verbal memory, and social cognition. Cognitive and/or behavioral impairment was also observed in 4 of 19 patients with early PLS (21.1%), indicating that such deficits may be present from the earliest disease stages. The frequency and pattern of impairment did not differ significantly from those observed in patients with predominant upper motor neuron ALS (PUMN‐ALS), the ALS phenotype most closely related to PLS. Differences were detected between patients with early PLS and those with PUMN‐ALS.

Previous studies investigating cognition and behavior in PLS have evaluated patients many years after symptom onset, typically once a definitive diagnosis had been established. In contrast, our study assessed patients much earlier, on average 26.2 months from onset in PLS and 19.5 months in PUMN‐ALS (*p* = 0.18), providing a unique opportunity to characterize cognitive and behavioral features in the earliest disease phases.

In a Dutch study of 30 PLS patients assessed after a mean disease duration of 16.3 years, 57% exhibited cognitive impairment, and 17% met diagnostic criteria for behavioral variant frontotemporal dementia [8]. A subsequent study from the same group [9], using the ECAS in 75 PLS patients, reported relatively preserved global cognitive function compared to ALS and PMA, but a significantly higher frequency of loss of sympathy/empathy (25% vs. 10%). We similarly identified this feature as a key behavioral manifestation, although at a lower frequency.

Other studies conducted after 9–12 years of disease duration have also documented cognitive and/or behavioral impairments in PLS, including deficits in verbal fluency and language, as well as apathy [20, 21].

At the time of their first visit to the ALS Center, our cohort of PLS patients showed poorer cognitive performance than matched healthy controls across multiple domains, including executive function, verbal and visual memory, visuo‐constructive abilities, attention/working memory, fluid intelligence, and social cognition (theory of mind). They also had higher scores on the depression and anxiety subscales of the HADS. These findings indicate that PLS, similar to ALS, is a multidimensional disorder, with cognitive and behavioral involvement already detectable in the early stages of the disease, thereby supporting observations made in more advanced phases [8]. Neuroimaging studies have shown early cortical involvement in probable PLS [22], and longitudinal PLS studies have documented progressive cortical change [23]. In addition to recent reports of frontotemporal involvement, there is emerging evidence of subcortical, thalamic, and hippocampal [24] as well as cerebellar [25] degeneration in PLS, which likely contribute to the multi‐domain cognitive and behavioral manifestations observed in this cohort.

When compared with matched PUMN‐ALS patients, individuals with PLS performed worse primarily in the executive domain, while exhibiting a comparable degree of behavioral impairment. However, the qualitative profile of behavioral symptoms differed: apathy, and to a lesser extent disinhibition and stereotyped behaviors, were more frequent in PUMN‐ALS. These observations align with previous findings [8] and may reflect distinct behavioral phenotypes within these two upper motor neuron–dominant disorders. Pseudobulbar affect is frequently observed in PLS; while traditionally linked to bilateral corticobulbar tract degeneration [26], cerebellar and frontotemporal components have also been implicated [27].

This study has some limitations. First, although the PLS cohort was relatively small, it represents a complete, systematically identified population derived from a prospective epidemiological registry. Moreover, while not all individuals from the original epidemiological series underwent assessment, those who were not evaluated differed from assessed participants only in terms of older age, and not in formal education level, thereby mitigating the risk of selection bias. Second, the number of age‐ and sex‐matched controls was limited; however, controls were matched to PLS cases at a 2:1 ratio using propensity score methods, which strengthens the robustness of between‐group comparisons. Third, functional status was quantified using the ALSFRS‐R rather than the recently developed and validated PLS‐specific Functional Rating Scale (PLSFRS) [6]; the PLSFRS was not yet available during most of the enrolment period.

In conclusion, our study demonstrates that a substantial proportion of individuals with PLS showed cognitive and behavioral impairments already at their early stages. Overall, their cognitive–behavioral profiles did not differ significantly from those of patients with the PUMN‐ALS phenotype, which closely resembles PLS in terms of motor involvement.

Our findings reinforce the view that PLS is a multidimensional disease that affects not only motor function, albeit limited to upper motor neurons, but also cognitive and behavioral domains from its earliest phases. By deepening our understanding of the nature of this rare condition, these results may help refine the clinical management of PLS and guide the design of future therapeutic trials.

## Author Contributions

**Andrea Calvo:** conceptualization, validation, writing – review and editing, writing – original draft. **Rosario Vasta:** conceptualization, formal analysis, writing – review and editing. **Enrico Matteoni:** conceptualization, writing – review and editing. **Anastasia Dei Giudici:** conceptualization, investigation, writing – review and editing. **Giorgio Pellegrino:** conceptualization, validation, writing – review and editing. **Umberto Manera:** conceptualization, validation, writing – review and editing. **Antonio Canosa:** conceptualization, validation, writing – review and editing. **Emilio Minerva:** conceptualization, validation, writing – review and editing. **Sara Cabras:** conceptualization, writing – review and editing. **Vita Passidomo:** conceptualization, investigation, writing – review and editing. **Gabriele Mora:** conceptualization, writing – original draft, writing – review and editing, validation, formal analysis. **Barbara Iazzolino:** conceptualization, data curation, investigation, visualization, writing – review and editing, validation, methodology. **Francesca Palumbo:** conceptualization, investigation, validation, writing – review and editing, data curation, methodology. **Alessandra Maccabeo:** conceptualization, writing – review and editing. **Adriano Chiò:** formal analysis, data curation, conceptualization, writing – original draft, funding acquisition, writing – review and editing, validation, resources. **Cristina Moglia:** conceptualization, investigation, validation, writing – review and editing.

## Funding

This work was supported by Ministero della Salute, RF‐2016‐02362405. Ministero dell'Università e della Ricerca, 2017SNW5MB, 20228N7573. Seventh Framework Programme, 259867. Horizon 2020 Framework Programme, 101017598. HORIZON EUROPE Framework Programme, 101137074. Agenzia di Ricerca per la Sclerosi Laterale Amiotrofica, DIG‐ALS.

## Conflicts of Interest

The authors declare no conflicts of interest.

## Supporting information


**Table S1:** Neuropsychological and behavioral tests administered, classified according to the main cognitive domain assessed.
**Table S2:** Characteristics of PLS patients included and not included in the study.
**Table S3:** Comparison cognitive and behavioral classification of early, probable and/definite PLS (*p* = 0.11).
**Table S4:** Comparison of cognitive and behavioral classification of PLS and matched PUMN‐ALS (*p* = 0.15).

## Data Availability

Anonymised data relating to this article will be made available by request from any qualified investigator, subject to approval from the Comitato Etico Territoriale Azienda Ospedaliero‐Universitaria Città della Salute e della Scienza.

## References

[1] A. Chiò , A. Calvo , C. Moglia , L. Mazzini , G. Mora , and PARALS study group , “Phenotypic Heterogeneity of Amyotrophic Lateral Sclerosis: A Population Based Study,” Journal of Neurology, Neurosurgery, and Psychiatry 82, no. 7 (2011): 740–746, 10.1136/jnnp.2010.235952. 21402743

[2] B. S. de Vries , E. M. J. de Boer , F. Brugman , et al., “Primary Lateral Sclerosis: Implications for Diagnostic Criteria From a Natural History Study in The Netherlands,” Neurology 104, no. 11 (2025): e213461, 10.1212/WNL.0000000000213461. 40388677 PMC12092547

[3] D. G. Lester , A. G. Thompson , K. Talbot , and M. R. Turner , “Progression and Life Expectancy in Primary Lateral Sclerosis,” Journal of Neurology, Neurosurgery, and Psychiatry 96, no. 10 (2025): 1008–1011, 10.1136/jnnp-2025-336037. 40379483 PMC12505104

[4] R. Vasta , E. Matteoni , G. Pellegrino , et al., “The Epidemiology of Primary Lateral Sclerosis: Results From a Population‐Based Cohort,” Annals of Neurology 99 (2025): 606–613, 10.1002/ana.78105. 41316718 PMC12954144

[5] M. R. Turner , R. J. Barohn , P. Corcia , et al., “Primary Lateral Sclerosis: Consensus Diagnostic Criteria,” Journal of Neurology, Neurosurgery, and Psychiatry 91, no. 4 (2020): 373–377, 10.1136/jnnp-2019-322541. 32029539 PMC7147236

[6] I. Lee , G. Jang , Y. K. K. Cheung , et al., “Primary Lateral Sclerosis Natural History Study: Primary Lateral Sclerosis Functional Rating Scale and Other Outcomes Assessment,” Annals of Neurology 99, no. 2 (2026): 418–428, 10.1002/ana.78056. 41020440 PMC12871045

[7] M. de Carvalho , M. C. Kiernan , S. L. Pullman , K. Rezania , M. R. Turner , and Z. Simmons , “Neurophysiological Features of Primary Lateral Sclerosis,” Amyotrophic Lateral Sclerosis and Frontotemporal Degeneration 21, no. suppl (2020): 11–17, 10.1080/21678421.2020.1837174. 33602011

[8] B. S. de Vries , L. A. Spreij , L. M. M. Rustemeijer , et al., “A Neuropsychological and Behavioral Study of PLS,” Amyotroph Lateral Scler Frontotemporal Degener 20, no. 5–6 (2019): 376–384, 10.1080/21678421.2019.1620284. 31134825

[9] B. S. de Vries , L. M. M. Rustemeijer , L. A. Bakker , et al., “Cognitive and Behavioural Changes in PLS and PMA: Challenging the Concept of Restricted Phenotypes,” Journal of Neurology, Neurosurgery, and Psychiatry 90 (2019): 141–147, 10.1136/jnnp-2018-318788. 30076267

[10] E. Finegan , J. Kleinerova , O. Hardiman , et al., “Pseudobulbar Affect: Clinical Associations, Social Impact and Quality of Life Implications—Lessons From PLS,” Journal of Neurology 272, no. 4 (2025): 266, 10.1007/s00415-025-12971-y. 40072589 PMC11903626

[11] V. Vacchiano , L. Bonan , R. Liguori , and G. Rizzo , “Primary Lateral Sclerosis: An Overview,” Journal of Clinical Medicine 13, no. 2 (2024): 578, 10.3390/jcm13020578. 38276084 PMC10816328

[12] A. Chiò , G. Mora , C. Moglia , et al., “Secular Trends of Amyotrophic Lateral Sclerosis: The Piemonte and Valle d'Aosta Register,” JAMA Neurology 74, no. 9 (2017): 1097–1104, 10.1001/jamaneurol.2017.1387. 28692730 PMC5710181

[13] K. Rascovsky , J. R. Hodges , D. Knopman , et al., “Sensitivity of Revised Diagnostic Criteria for the Behavioural Variant of Frontotemporal Dementia,” Brain 134, no. Pt 9 (2011): 2456–2477, 10.1093/brain/awr179. 21810890 PMC3170532

[14] M. J. Strong , S. Abrahams , L. H. Goldstein , et al., “Amyotrophic Lateral Sclerosis ‐ Frontotemporal Spectrum Disorder (ALS‐FTSD): Revised Diagnostic Criteria,” Amyotrophic Lateral Sclerosis and Frontotemporal Degeneration 18, no. 3‐4 (2017): 153–174, 10.1080/21678421.2016.1267768. 28054827 PMC7409990

[15] K. B. Kortte , M. D. Horner , and W. K. Windham , “The Trail Making Test, Part B: Cognitive Flexibility or Ability to Maintain Set?,” Applied Neuropsychology 9 (2002): 106–109, 10.1207/S15324826AN0902_5. 12214820

[16] E. Beeldman , J. Raaphorst , M. Klein Twennaar , M. de Visser , B. A. Schmand , and R. J. de Haan , “The Cognitive Profile of ALS: A Systematic Review and Meta‐Analysis Update,” Journal of Neurology, Neurosurgery, and Psychiatry 8 (2016): 611–619, 10.1136/jnnp-2015-310734. 26283685

[17] E. Beeldman , J. Raaphorst , M. Klein Twennaar , et al., “The Cognitive Profile of Behavioural Variant FTD and Its Similarities With ALS: A Systematic Review and Meta‐Analysis,” Journal of Neurology, Neurosurgery, and Psychiatry 89 (2018): 995–1002, 10.1136/jnnp-2017-317459. 29439163

[18] B. Poletti , F. Solca , L. Carelli , et al., “Cognitive‐Behavioral Longitudinal Assessment in ALS: The Italian Edinburgh Cognitive and Behavioral ALS Screen (ECAS),” Amyotroph Lateral Scler Frontotemporal Degener 19, no. 5–6 (2018): 387–395, 10.1080/21678421.2018.1473443. 29804470

[19] B. Poletti , E. N. Aiello , F. Solca , et al., “Diagnostic Properties of the Italian ECAS Carer Interview (ECAS‐CI),” Neurological Sciences 44, no. 3 (2023): 941–946, 10.1007/s10072-022-06505-x. 36417015 PMC9925466

[20] E. Finegan , S. L. H. Shing , R. H. Chipika , et al., “Extra‐Motor Cerebral Changes and Manifestations in Primary Lateral Sclerosis,” Brain Imaging and Behavior 15, no. 5 (2021): 2283–2296, 10.1007/s11682-020-00421-4. 33409820

[21] A. Meoded , J. Y. Kwan , T. L. Peters , et al., “Imaging Findings Associated With Cognitive Performance in Primary Lateral Sclerosis and Amyotrophic Lateral Sclerosis,” Dementia and Geriatric Cognitive Disorders Extra 3, no. 1 (2013): 233–250, 10.1159/000353456. 24052798 PMC3776403

[22] E. Finegan , S. Li Hi Shing , W. F. Siah , et al., “Evolving Diagnostic Criteria in Primary Lateral Sclerosis: The Clinical and Radiological Basis of ‘Probable PLS’,” Journal of the Neurological Sciences 417 (2020): 117052, 10.1016/j.jns.2020.117052. 32731060

[23] M. Tahedl , S. Li Hi Shing , E. Finegan , et al., “Propagation Patterns in Motor Neuron Diseases: Individual and Phenotype‐Associated Disease‐Burden Trajectories Across the UMN‐LMN Spectrum of MNDs,” Neurobiology of Aging 109 (2021): 78–87, 10.1016/j.neurobiolaging.2021.04.031. 34656922

[24] E. Finegan , S. Li Hi Shing , R. H. Chipika , et al., “Thalamic, Hippocampal and Basal Ganglia Pathology in Primary Lateral Sclerosis and Amyotrophic Lateral Sclerosis: Evidence From Quantitative Imaging Data,” Data in Brief 29 (2020): 105115, 10.1016/j.dib.2020.105115. 32055654 PMC7005372

[25] E. Finegan , W. F. Siah , S. Li Hi Shing , R. H. Chipika , O. Hardiman , and P. Bede , “Cerebellar Degeneration in Primary Lateral Sclerosis: An Under‐Recognized Facet of PLS,” Amyotroph Lateral Scler Frontotemporal Degener 23, no. 7–8 (2022): 542–553, 10.1080/21678421.2021.2023188. 34991421

[26] M. Tahedl , E. L. Tan , W. F. Siah , et al., “Radiological Correlates of Pseudobulbar Affect: Corticobulbar and Cerebellar Components in Primary Lateral Sclerosis,” Journal of the Neurological Sciences 451 (2023): 120726, 10.1016/j.jns.2023.120726. 37421883

[27] P. Bede and E. Finegan , “Revisiting the Pathoanatomy of Pseudobulbar Affect: Mechanisms Beyond Corticobulbar Dysfunction,” Amyotrophic Lateral Sclerosis & Frontotemporal Degeneration 19, no. 1–2 (2018): 4–6, 10.1080/21678421.2017.1392578. 29092641

