# Supplementary material for: Early Cognitive and Behavioral Changes in Primary Lateral Sclerosis: A Population‐Based Study
Source: Eur J Neurol. 2026 Jul 31;33(8):e70720. doi: 10.1111/ene.70720 (PMC13428224; doi:10.1111/ene.70720)
Supplement: Supplementary file 1 — Table S1: Neuropsychological and behavioral tests administered, classified according to the main cognitive domain assessed. Table S2: Characteristics of PLS patients included and not included in the study. Table S3: Comparison cognitive and behavioral classification of early, probable and/definite PLS (p = 0.11). Table S4: Comparison of cognitive and behavioral classification of PLS and matched PUMN‐ALS (p = 0.15). [file ENE-33-e70720-s001.docx]

**Table S1**. Neuropsychological and behavioral tests administered, classified according to the main cognitive domain assessed

| **Domains** | **Tests** |
| --- | --- |
| Executive functions | Letter Fluency test (FAS) |
|  | Category Fluency Test (CAT) |
|  | Trail Making Test B-A (TMT B-A) |
|  | Frontal Assessment Battery (FAB) |
|  | ECAS Executive Function score |
|  | ECAS Verbal Fluency score |
| Verbal memory | Rey Auditory Verbal Learning Test, Immediate Recall (RAVL-IR) |
|  | Rey Auditory Verbal Learning Test, delayed Recall (RAVL-DR) |
|  | Babcock Story Recall Test, Immediate Recall (BSRT-IR) |
|  | Babcock Story Recall Test, Delayed Recall (BSRT-DR) |
|  | ECAS Memory score |
| Language | Token test (up to 2016) |
|  | Battery for the Analysis of Aphasic Deficits (semantic systems tests 7 and 8) (up to 2016) |
|  | Boston Naming Test (from 2016) |
|  | ECAS Language score (from 2016) |
| Visual Memory | Rey-Osterrieth Complex Figure Test, differed recall (ROCF-DR) |
| Visuoconstructive abilities | Rey-Osterrieth Complex Figure Test, Immediate Recall (ROCF-IR) |
|  | Clock Drawing Test (Clock) |
|  | ECAS Visuospatial Abilities score |
| Attention/working memory | Digit Span Forward (FW) |
|  | Digit Span Backward (BW) |
| Psychomotor speed | Trail Making Test A (TMT A) |
| Fluid intelligence | Raven’s Colored Progressive Matrices (CPM47) |
| Cognitive flexibility | Trail Making Test B (TMT B) |
| Theory of mind (social cognition) | Story-based Empathy Task (SET) (from 2018) |
| Behavior | Frontal Systems Behavior Scale (FrSBe) |
|  | ECAS Behavior score |
|  | Frontal Behavioural Inventory (FBI) |
| Non-ALS | Mini Mental State Examination (MMSE) |

**Legend:** Tests are grouped by the principal neuropsychological domain they assess. The battery was selected according to the Diagnostic Criteria for the behavioral variant of Frontotemporal Dementia and the ALS-FTD Consensus Criteria. Where a test was used only during a defined time window, or replaced another test over the study period, the relevant years are indicated in parentheses. ECAS, Edinburgh Cognitive and Behavioural ALS Screen; FBI, Frontal Behavioural Inventory; FrSBe, Frontal Systems Behavior Scale; MMSE, Mini Mental State Examination.

**Table S2**. Characteristics of PLS patients included and not included in the study

|  | Patients included in the study  n=32 | Patients not included in the study  n=25 | p |
| --- | --- | --- | --- |
| Age at test (years, SD) | 60.5 (8.5) | 67.3 (9.6) | 0.04 |
| Education (median, years, SD) | 10.2 (3.6) | 9.3 (4.2) | 0.07 |
| Sex (female) | 16 (50.4%) | 20 (80%) | 0.04 |
| Onset to test time (median months, SD) | 24.6 (19.5) | 20.2 (21.3) | 0.36 |
| Site of onset (spinal) | 29 (90.6%) | 17 (68%) | 0.07 |
| ALSFRS-R score (median) | 40.6 (4.5) | 42.6 (5.1) | 0.28 |
| ∆ALSFRS-R (points/month), median, SD | 0.38 (0.30) | 0.31 (0.31) | 0.67 |

**Table S3**. Comparison cognitive and behavioral classification of early, probable and /definite PLS (p=0.11)

|  | Early PLS (n=19) | Probable PLS (n=10) | Definite PLS (n=3) |
| --- | --- | --- | --- |
| PLS-CN | 15 (78.9%) | 5 (50%) | 2 (66.7%) |
| PLS-bi | 2 (10.5%) | 0 | 0 |
| PLS-ci | 1 (5.2%) | 5 (50%) | 1 (33.3%) |
| PLS-cbi | 1 (5.2%) | 0 | 0 |

**Table S4**. Comparison of cognitive and behavioral classification of PLS and matched PUMN-ALS (p=0.15)

|  | PLS (n=32) | PUMN-ALS (n=64) |
| --- | --- | --- |
| PLS-CN /ALS-CN | 22 (68.8%) | 44 (68.8%) |
| PLSbi /ALSbi | 3 (9.4%) | 10 (15.6%) |
| PLSci /ALSci | 6 (18.8%) | 3 (4.7%) |
| PLScbi /ALSci | 1 (3.1%) | 6 (9.4%) |
| FTD | 0 | 1 (1.6%) |
